# Healthful vs. Unhealthful Plant-Based Restaurant Meals

**DOI:** 10.3390/nu17050742

**Published:** 2025-02-20

**Authors:** Kim A. Williams, Amy M. Horton, Rosella D. Baldridge, Mashaal Ikram

**Affiliations:** 1 Department of Internal Medicine, School of Medicine, University of Louisville, 550 South Jackson Street, Louisville, KY 40202, USA; 2 Office of Community Engagement, University of Louisville, Louisville, KY 40202, USA; 3 Department of Medicine (Cardiology), University of Chicago—Endeavor Health System, 2650 Ridge Ave., Evanston, IL 60201, USA; mashaal.ikram12@gmail.com

**Keywords:** restaurants, vegan, vegetarian, healthful, unhealthful, refined grains, saturated fat, fried food

## Abstract

*Background:* Vegan/vegetarian (VEG) restaurants and VEG options in omnivore (OMNI) restaurants may serve unhealthful plant-based food that may be more harmful than a typical American diet. *Methods:* A sample of 561 restaurants with online menus were analyzed over a 3-year period. Each plant-based menu entrée was counted, up to a maximum of ten entrées per restaurant, meaning that a restaurant customer could select from ten or more healthful plant-based choices. Entrées containing refined grains (e.g., white rice and refined flour), saturated fat (e.g., palm oil and coconut oil), or deep-fried foods were counted as zero. *Results:* We evaluated 278 VEG and 283 OMNI restaurants. A full menu (10 or more plant-based entrées) was available in 59% of the VEG, but only 16% of the OMNI (*p* < 0.0001). Zero healthful options occurred in 27% of OMNI, but only 14% of VEG (*p* = 0.0002). The mean healthy entrée count for all restaurants was 3.2, meaning that, on average, there were only about three healthful plant-based choices of entrées on the menu, significantly more in VEG (4.0 vs. 2.4 *p* < 0.0001). The most common entrée reduction was for refined grains (e.g., white flour in veggie-burger buns or white rice in Asian entrées, n = 1408), followed by fried items (n = 768) and saturated fat (n = 318). VEG restaurants had a significantly higher frequency of adequate VEG options (≥7 options, 24% vs. 13%, *p* = 0.0005). *Conclusions:* Restaurants listed as VEG have a slightly higher number of healthful entrées than OMNI restaurants, which offer more limited vegan/vegetarian options. Given the published relationship between unhealthful dietary patterns, chronic illness, and mortality, we propose that detailed nutrition facts be publicly available for every restaurant.

## 1. Introduction

Plant-based eating patterns—often called vegan and/or vegetarian (VEG)—are growing in the United States and around the world. In the USA, the vegan food market reached approximately USD 11 billion as of 2023, with the meat-free and dairy alternative segments accounting for about USD 2.33 billion and USD 2.8 billion, respectively. Vegan market growth has been projected at 12% annually through 2030 [1].

This commercial growth has led to more VEG food options in grocery stores, an increase in dedicated VEG restaurants, and a growing number of VEG-labeled options in non-vegetarian (OMNI) restaurants. For example, more than 30,000 vegan and vegan-friendly restaurants are listed on platforms such as HappyCow [1]. Four main categories of VEG patrons fuel this increase: those driven by ethical concerns (anti-animal cruelty), environmental concerns (planetary sustainability), health-related reasons (reduced chronic disease risk), and/or religious beliefs. However, only a small minority—less than 2% of respondents in one National Health Interview Survey study—reported adopting a vegetarian or vegan diet for health reasons [2]. In fact, it has been estimated that 63% of the growth in plant-based eating is attributable to OMNI patrons rather than members of the VEG community.

Despite this growth, evidence suggests that some plant-based foods can be unhealthy [3,4]. For instance, juices and sweetened beverages, refined grains, potatoes/fries, and sweets may contribute to adverse health outcomes. Consumption of these items—and of animal foods that typically contain cholesterol and saturated fat—is recommended to be reduced or avoided according to the American College of Cardiology/American Heart Association (ACC/AHA) Guideline on the Primary Prevention of Cardiovascular Disease [5]. Similarly, the World Cancer Research Fund/American Institute for Cancer Research (WCRF/AICR) has compiled evidence linking fat and refined grain consumption to cancer risk [6], and the International Agency for Research on Cancer (IARC) has summarized data that associate the ingestion of fried foods and the resultant acrylamides with cancer production [7].

Many plant-based foods are designed and processed to appeal to popular tastes and may contain high levels of sodium, saturated fat, refined grains (such as white flour or white rice), and added sugar, or are deep fried. Consumption of these foods has been associated with increased mortality [8] and a higher risk of coronary heart disease—even when compared to a general American diet [3].

The primary purpose of this study was to determine how often a restaurant patron, seeking vegan options at an establishment advertised online as offering VEG choices, is presented with (a) a variety of healthful options (defined as 10 or more choices) and (b) options free from refined grains, saturated fat, or fried foods. A secondary aim was to assess the frequency with which detailed nutritional breakdowns are available online. Such information would allow consumers to identify unhealthy choices—those high in saturated fat or sodium or with a high sugar-to-fiber ratio.

## 2. Methods

In accordance with 45 CFR §46.102(f), this nutritional evaluation study was not submitted for institutional review-board approval or preregistered (https://clinicaltrials.gov), because it involved publicly available data and did not gather any patient data.

### 2.1. Study Design

Over a 3-year period, members of our assessment team visited a convenience sample of 561 restaurants purported to have vegan offerings, typically located close to the venue of a medical conference. This sample included 196 cities in 37 countries, mostly from USA (n = 373, in 26 states and the District of Columbia), Australia (n = 48), France (n = 29), England (n = 17), Germany (n = 16), Italy (n = 13), and Spain (n = 10) (see Appendix A for full list).

### 2.2. Inclusion and Exclusion Criteria

To qualify for inclusion in the sample, the restaurant was required to have an internet listing on a proprietary commercially available online resource, such as Caviar (https://www.trycaviar.com), DoorDash (https://www.doordash.com), Grubhub (https://www.grubhub.com), Happy Cow (https://www.happycow.net), search engines such as Google, or Uber Eats (https://www.ubereats.com/), and a confirmed online menu available for analysis containing a detailed listing of their non-dairy vegetarian (“vegan”) options. We excluded restaurants that only provided vegan options ad hoc, i.e., changing the menu items listed “upon request”, but are not listed online as having plant-based menu items. For each restaurant, we also searched online for the availability of nutrition facts, which would allow more in-depth evaluation of healthfulness, as mandated in the USA for any franchise with more than 20 locations [9].

### 2.3. Scoring of Healthy vs. Unhealthy Entrées

We analyzed the menu’s entrée items, rather than appetizers, side salads, side dishes, desserts, or beverages, compiling the name and analyzing the description of all items listed as “vegan”. Each entrée was given one point, up to a maximum of ten entrées per restaurant, typically using the top entry in each entrée section. For example, a VEG Chinese restaurant may have sections entitled the following: Vegan Meat Alternatives, Vegetable-Based Entrées, Noodle and Rice Dishes, Rice Dishes, and Tofu and Bean Curd Dishes; in this case of 5 sections, the first 2 entries in each category comprised the 10 selections for analysis.

Like one prior published analysis [3], an entrée was considered unhealthful and was deducted from the healthful entrée total if it contained either refined grains (e.g., white rice and refined flour), saturated fat (e.g., palm oil, coconut oil, coconut milk, or coconut cream), or deep-fried foods. The final restaurant healthy entrée total ranged from 0 to 10 (i.e., 10 entrée options with no removals for unhealthful ingredients). If an entrée with an unhealthy ingredient had a healthier option listed on the menu, e.g., brown rice instead of white rice, whole-grain bun vs. white-flour bun, or steamed tofu vs. fried tofu, the healthier option was credited to that entrée.

### 2.4. Refined Grains

Refined grains, i.e., processed to remove the germ (concentrated nutrients) and outer husk (bran fiber), leaving the endosperm (starchy middle), results in a higher glycemic index no longer balanced by fiber. Consumption of foods containing refined grains is associated with higher weight gain, dyslipidemia, cardiovascular disease, cancer, and mortality [6,10]. When noted, these were scored as −1. The presence of refined grains was determined by taste and texture, written description, or visual evidence in online images (e.g., baguette or a white bread veggie-burger or veggie-dog bun).

### 2.5. Saturated Fat

The presence of saturated fat was scored as −1 if the entrée listed cocoa butter, palm oil, coconut oil, or coconut milk, including both Impossible^TM^ meat (6 g per serving, https://www.fooddive.com/news/impossible-foods-reformulates-less-fat-than-beef/630756/ accessed on 1 February 2025) and Beyond Meat^TM^ (5 g per serving, https://www.beyondmeat.com/en-US/products/the-beyond-burger?variant=beyond-burger accessed on 1 February 2025). However, on 18 April 2024, Beyond Meat^TM^ announced a change from coconut oil to avocado oil, which reduced the saturated fat per serving to 2 g (https://www.beyondmeat.com/en-US/press/beyond-iv-the-fourth-generation-of-the-beyond-burger-and-beyond-beef-debuts-at-grocery-stores-across-the-u-s-including-at-walmart-and-kroger# accessed on 1 February 2025). Menu items entered after this date were not downgraded for saturated fat for Beyond Meat^TM^.

Vegan cheeses are generally at or below the recommended acceptable level of 10% of calories from 3.7 g of saturated fat [11], due to small serving size, given its use as a condiment (e.g., cheeseburger), or the use of low-saturated fat cheese (e.g., oat milk- or pea protein-derived cheese), which was usually not specified.

When identified on the menu, the nutritional components of other brands of plant-based meats were investigated to ensure a low saturated fat content. For example, LaVie bacon in Great Britain contains 1.3 g of saturated fat per 100 g serving, but 2000 mg of sodium (https://www.laviefoods.com/en/nutrition/ accessed on 1 February 2025).

### 2.6. Fried Foods

The health concerns surrounding the consumption of fried food center on its markedly increasing fat content and carcinogenic potential [12,13,14,15]. Given these concerns and epidemic planetary levels of obesity and metabolic syndrome, the presence of fried foods in an entrée was assigned a −1 score. However, for this study, stir-fried and pan-fried were not scored as deep-fried due to the lower oil content, but “battered” and “crispy” items were considered deep-fried, unless otherwise specified on the menu.

### 2.7. Statistics

Data on entrée counts and reductions are presented as mean and standard deviation. Comparison of means was performed with XLSTAT version 2024.1—Life Sciences, with the null hypothesis that there was no difference between the number of healthy options at VEG and OMNI restaurants. Normality of the distribution of entrée counts was evaluated with the Shapiro–Wilk test, and each group’s result was found to not follow a normal distribution. Therefore, the Mann–Whitney test was used to determine statistical significance of comparison of means of independent samples with a two-tailed *t*-test. Chi-square with one degree of freedom was utilized for frequency comparisons between VEG and OMNI restaurants. A *p*-value of < 0.05 was considered statistically significant.

## 3. Results

### 3.1. Restaurant Distribution

A total of 561 (278 VEG and 283 OMNI) restaurants were analyzed. In two restaurants, animal products were erroneously labeled as “vegan”: one VEG restaurant listed dairy (“non-vegan”) cheese on four items despite labeling as “vegan”, and one OMNI restaurant mistakenly labeled an entrée of “spicy stew of pork, brisket, turkey, veggies” as vegan on both the written and online menus. These menu items were excluded or censured from the analysis.

When the 373 United States of America (USA) and 188 non-USA restaurants were compared, there was no significant difference in the total available entrées per restaurant (USA = 6.6; non-USA = 6.2; *p* = 0.238), but the average number of healthful entrées trended slightly higher in the USA (3.4 vs. 2.9, *p* = 0.075).

Complete nutritional disclosure from online nutrition facts (including sodium content, calories, total and added sugar, or quantitation of total and saturated fat) was available in only 28 OMNI and 5 VEG restaurants (10% vs. 2%, *p* = 0.0001).

### 3.2. Entrée Healthfulness

There was an average of 4.5 healthful vegan entrées from OMNI restaurants (1276 out of a possible 2830 entrées), and 8.4 healthful entrées from VEG restaurants (2333 out of a possible 2780, *p* < 0.0001 vs. OMNI). A full menu (i.e., 10 or more plant-based-option entrées) was available in 59% of the VEG, but only 16% of the OMNI restaurants (*p* < 0.0001).

**Figure 1:**
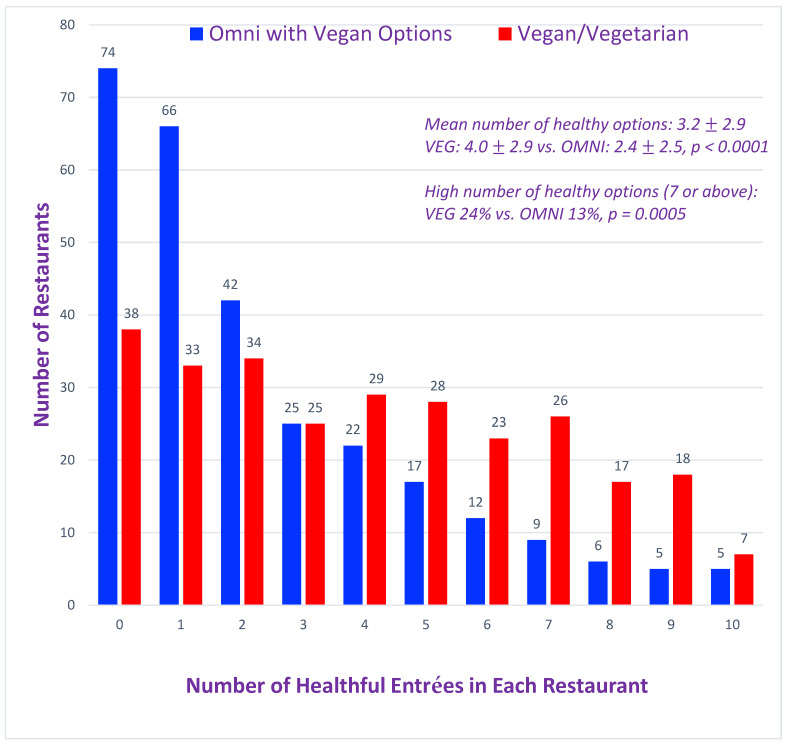
Frequency distribution of restaurant scores. Out of 561 restaurants, 278 were listed as vegan/vegetarian, and 283 were omnivore (OMNI) restaurants reporting to have vegan/vegetarian (VEG) options on the menu. The average healthfulness score was 3.2 ± 2.9, indicating just over 3 healthful plant-based options, defined as no animal products (i.e., zero cholesterol content), refined grains, excessive saturated fat or fried items. The OMNI restaurants scored slightly but significantly lower than VEG (*p* < 0.0001). There were more high-scoring (7 or above) VEG restaurants (*p* = 0.0005).

The mean healthfulness score of all restaurants was 3.2 ± 2.9, meaning that on average there were only about three healthful plant-based choices for entrées on the menu (Figure 1). VEG restaurants had slightly but significantly more healthful entrées than the OMNI restaurants (4.0 ± 2.9 vs. 2.4 ± 2.5, *p* < 0.0001). Out of 3609 entrées analyzed, there were only 1120 VEG and 676 OMNI healthful entrées (48% vs. 53%, *p* = 0.0998). VEG restaurants had the greatest number of healthy options, defined as a restaurant score of ≥7 out of the maximum 10, 24% vs. 13% for OMNI, *p* = 0.0005. A maximum entrée score of 10, i.e., no refined grains, no saturated-oil use, and no fried components, was found in only 2% of the restaurants. Zero healthful options were found in 26% (74/283) of OMNI, but only 14% (38/278) of VEG restaurants (*p* = 0.0002).

### 3.3. Score Reduction Analyses

**Figure 2:**
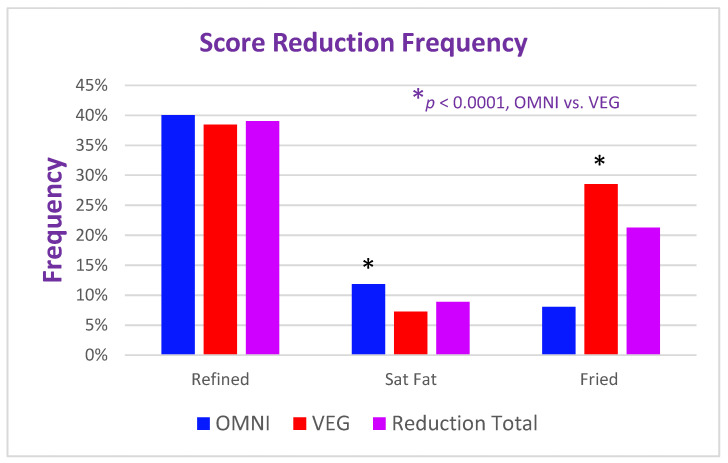
When normalized for the number of observations, 1276 for OMNI and 2333 for VEG, score reduction for refined grains (Refined) was the most common (e.g., non-whole-grain pasta, white flour in bread or burger buns, or white rice in Indian or Asian entrées) 40% for OMNI and 38% for VEG, *p* = NS. Reduction for saturated fat was more frequent in OMNI (12% vs. 7%, *p* = 0.0001) and reduction for fried items occurred more frequently in VEG (29% vs. 8%, *p* < 0.0001).

A total of 2497 entrées had reduced scores, including 1408 reduced for refined grains, 321 for saturated fat, and 768 for fried foods. VEG restaurants had a higher frequency of score reductions than OMNI (1732 in 2333 entrées vs. 765 in 1276, *p* = 0.0001). As in Figure 2, when normalized for the number of entrées, score reduction for refined grains was the most common (e.g., non-whole-grain pasta, white flour in bread or burger buns, or white rice in Indian or Asian entrées) 40% for OMNI and 38% for VEG, *p* = NS. Reduction for saturated fat was more frequent in OMNI (12% vs. 7%, *p* = 0.0001), and reduction for fried items occurred more frequently in VEG (28% vs. 8%, *p* < 0.0001).

## 4. Discussion

This is the first published study to evaluate the healthfulness of plant-based restaurant offerings—adhering to the 2019 ACC/AHA Primary Prevention Guideline recommendations [5]—across all six continents where restaurants exist (i.e., excluding Antarctica). Our convenience sample of restaurants purported to serve plant-based (“vegan”) options revealed several central findings:

- There are many unhealthful plant-based foods labeled as “vegan” in both OMNI and VEG restaurants.
- Slightly more healthful options are available in VEG than in OMNI restaurants.
- Refined-grain use is common and represents the largest source of unhealthfulness in entrées.
- Healthful options are marginally more likely to be found in USA restaurants compared with non-USA restaurants.

Full disclosure of nutritional content—specifically sodium, sugar, and saturated fat—is not widely available. However, such disclosure is more frequent in OMNI restaurants than in VEG restaurants, largely due to the presence of plant-based options in USA restaurant chains that are mandated to disclose nutrition information.

These results are not unexpected, as the restaurants must respond to the desires of their customers. Customers are often limited by multiple barriers to healthy nutrition habits, which have been recently analyzed using artificial intelligence [16]. These included cultural and traditional habits; taste and familiarity; accessibility and affordability; nutritional-adequacy concerns; social norms and peer pressure; marketing and industry influences; lack of education; emotional attachments; convenience and time constraints; and psychological resistance to change. The authors pointed out that restaurants are “not in the healthcare business” and respond to market forces, since “they will not sell what we won’t buy”.

One previous, smaller publication on a similar topic [17] examined the menus of 73 restaurant chains with complete online nutrition information. Using the 2017 AHA criteria and a scoring system similar to ours (assigning either 0 or 1 point per entrée), they evaluated meals for the following criteria:

- Approximately 600 calories or fewer per meal;
- No more than 35% of calories from fat;
- Less than 5 g of saturated fat;
- Zero grams of trans fat;
- Cholesterol below 75 mg per meal;
- Sodium less than 700 mg per meal;
- At least 10 g of protein per meal;
- At least 5 g of fiber per meal.

They found that fewer than 20% of meals met the saturated fat and sodium criteria. In total, 22% of restaurant meals met zero to one AHA criteria, 50% met two to four criteria, 20% met five to six criteria, and only 8% met all seven criteria. They called for improvements in the healthiness of restaurant meals. However, that study was unable to evaluate meals using the more rigorous nutrition recommendations of the 2019 ACC/AHA Guidelines, and it did not assess fried foods or refined grains, which are known to accelerate chronic disease and cardiovascular risk.

In the United States, cardiovascular disease remains the leading cause of death. Nutrition-mediated acute and chronic diseases contribute significantly to rising healthcare costs, loss of life, and reduced economic and personal productivity [18]. Although the food and restaurant industries are not primarily in the healthcare business, their long-term sustainability depends on consumers’ purchasing habits. Consumer choices are heavily influenced by habit, culture, and television marketing of appealing yet unhealthy food—a phenomenon some have termed “marketing mortality” [19]. This influence disproportionately affects individuals with socioeconomic disadvantages [20,21]. For example, a recent study found that more than one-third of American families earning less than USD 9000 per year watched over five hours of television daily, compared with only 1% of families earning USD 150,000 per year [20]. Such media exposure has a particularly strong influence on economically disadvantaged children [21]. The percentage of children watching television for one hour or more per day increases from age 2 to 9 years, reaching 85% for children of low-educated mothers compared with 61% for children of highly educated mothers.

### 4.1. Healthful vs. Unhealthful Vegan/Vegetarian Food: Helpful or Unhelpful?

The recent ACC/AHA Cardiovascular Disease Primary Prevention Guidelines [5] recommend reducing consumption of foods that increase the risk of cardiac events and/or mortality. Observational data from the PURE trial indicate that, despite the risks associated with saturated fat, replacing refined carbohydrates with either saturated or unsaturated fats can reduce stroke and mortality [22]. Furthermore, a reduction in dietary sodium was shown to lower blood pressure and reduce cardiovascular events in the DASH trial and the Trials of Hypertension Prevention (TOHP) [23,24]. High consumption of sodium (>2000 mg daily), red meat (>14 g/d), sugar-sweetened beverages, and processed red meat has been associated with cardiovascular death and increased all-cause mortality in the NHANES [25].

A prospective cohort study of USA healthcare professionals [26] demonstrated that replacing animal protein with vegetable protein—thereby reducing the ingestion of compounds associated with increased cardiovascular and cancer risk, such as cholesterol, saturated fat, insulin-like growth factor (IGF-1), heme iron [27], advanced glycation end products (AGEs), polyaromatic hydrocarbons, and heterocyclic amines produced during high-temperature cooking [28]—is associated with reduced mortality. Additionally, precursors of trimethylamine-N-oxide (e.g., choline, betaine, carnitine, and phosphatidylcholine) have been linked to coronary artery disease, chronic kidney disease, and heart failure mortality; a plant-based diet may reduce these precursors through wholesale changes in the gastrointestinal microbiome [29]. Conversely, plant-protein consumption is associated with a 10% reduction in mortality for every 3% energy increment that replaces animal protein [26]. Moreover, consumption of sugar-sweetened and artificially sweetened beverages increases the risk of type 2 diabetes and cardiovascular events, with one daily serving linked to a 20% increase in diabetes risk [30]. Consumption of sugar exceeding 10% of daily calories has been associated with increased mortality [31].

The REGARDS (REasons for Geographic and Racial Differences in Stroke) study [32] found that the “Southern” dietary pattern—characterized by high intake of animal products and foods that scored negatively in our evaluation—is associated with a 56% higher risk of heart disease, a 50% increase in chronic kidney disease, and a 30% higher risk of stroke. Additionally, there is clear evidence that some plant-based foods are unhealthful [3]. Examples include fruit juices (which lack fiber), sugar- and artificially sweetened beverages, refined grains, potatoes/fries, and sweets. This pattern of eating may contribute to coronary disease development at a higher rate than the consumption of animal products [3].

### 4.2. Fried Food: Waste It or Just “Waist” It?

Deep frying a raw potato increases its fat content from 0.2 g per 100 g to 13.2 g—a 66-fold increase [10]. In cases where frying oil is rich in monounsaturated or polyunsaturated fats (e.g., olive oil or canola oil) and used in moderation, the patron’s lipid profile may improve [13]. However, for overweight or obese individuals, such frying may lead to weight gain and higher triglycerides. Enhanced taste from frying can also lead to increased food consumption due to dopamine release [33,34], thereby contributing to the rising global obesity rates [35,36] and systemic hypertension [37].

One isolated study reported that frying vegetables in olive oil is healthier than boiling them and may help prevent cancer, diabetes, and vision loss [38]. Frying may preserve vitamin C and B vitamins and could even increase the fiber content in potatoes by converting starch into resistant starch [39]. However, frying certain vegetables (such as in French fries or potato chips) may result in the formation of acrylamides via the Maillard reaction, which has been associated with cancer formation in animal models, though the evidence in humans is less clear [14,15]. Additionally, high-heat cooking forms AGEs, which are associated with inflammation, oxidative stress, and chronic diseases such as type 2 diabetes mellitus [40]. Finally, frying in repeatedly heated oils can produce trans fats, which are linked to adverse cardiovascular events [41].

### 4.3. Refined, but Against the Grain

We assigned negative scores to refined grains in our analysis based on their association with increased risk of death and disease compared to whole grains. A recent meta-analysis of 64 studies [10] demonstrated that whole-grain intake—with its high fiber content—is associated with a reduced risk of coronary heart disease; cardiovascular disease; various cancers; and overall mortality, including deaths from respiratory and infectious diseases, diabetes, and non-cardiovascular/non-cancer causes. The authors propose that replacing refined grains with whole grains could substantially reduce the burden of chronic disease if widely adopted. In contrast, international studies have indicated that consumption of refined grains is associated with increased total mortality, a higher incidence of major cardiovascular events, and elevated systolic blood pressure [8,42].

### 4.4. Saturated with Controversy

In 2014, the lay press widely reported that advice to avoid saturated fat was incorrect [43], challenging established science. However, subsequent reanalyses [44,45,46] and compilations of the data [47] have reinforced the link between saturated fat consumption and dyslipidemia, cardiac events, and mortality, despite some data suggesting that saturated fat is less harmful than refined carbohydrates [44]. Moreover, saturated fats can activate the inflammasome via toll-like receptor 4 (TLR4) signaling, leading to increased production of inflammatory mediators, whereas unsaturated fats do not exhibit this effect [48]. Higher intakes of saturated fat may also impair insulin signaling, contributing to insulin resistance, hepatic steatosis, obesity, and type 2 diabetes mellitus [49]. Diets high in saturated fats tend to be more energy dense and less satiating, which can promote weight gain [50]. In contrast, unsaturated fats—particularly omega-3 fatty acids—are neuroprotective and offer cognitive benefits relative to saturated fat intake [40]. There is also evidence that diets high in saturated fats disrupt the gut microbiome and increase gut permeability, while unsaturated fats may have beneficial effects on gut flora [37].

## 5. Limitations

Although this was an international study, most of the restaurants evaluated were in the USA and Westernized countries. This may limit the generalizability of our findings to restaurants in other regions. Our sampling was non-random and based primarily on proximity to the investigators’ homes, workplaces, and travel and urban medical meetings, which could introduce bias.

Moreover, our methodology did not permit personal sampling of every menu item. We relied on menu descriptions and available images to determine the use of refined grains. Similarly, assessing saturated fat content (specifically, whether it exceeded 10% of daily calories per serving) was often inferred rather than measured, given the limited availability of online nutritional information. In some cases, branded vegan products with known nutritional compositions—such as Impossible Meat™ (6 g per serving), Beyond Meat™ (5 g per serving), or Just Egg (0 g per serving)—were used in our grading. Notably, on 18 April 2024, Beyond Meat™ announced a switch from coconut oil to avocado oil, a change that should be associated with reduced LDL cholesterol and cardiac events [38]. Menu items containing Beyond Meat™ entered after this date were not downgraded for saturated fat. Lastly, due to incomplete nutritional information online, we could not quantitatively assess portion size or calories, levels of sodium, total fat, saturated fat, sugar, or sugar-to-fiber ratios.

## 6. Conclusions

In a very large international, although predominantly “Westernized”, evaluation of restaurants, healthful plant-based options are limited in OMNI restaurants but are slightly more available in VEG establishments, particularly in the US, compared with non-USA settings. Many menu items are high in refined grains—such as veggie-burger buns, white rice, and refined-grain pasta—and often include fried options and high levels of saturated fat, predominantly from coconut oil.

There is minimal transparency in the disclosure of nutritional facts that would help health-conscious patrons distinguish between healthful and unhealthful plant-based items. Since most restaurants do not provide detailed information on portion size, calories, sodium, total fat, saturated fat, total sugar, or added sugar content, even knowledgeable consumers may struggle to make informed choices. Given the well-established relationship between unhealthful dietary patterns, chronic illness, and mortality—and the relative ease with which nutritional information could be provided—we propose that detailed nutrition facts be made publicly available for every restaurant.

We recommend that the Food and Drug Administration in the USA (FDA) and international regulatory bodies expand requirements for nutritional disclosure beyond franchises with 20 or more locations. We suggest that restaurants reevaluate the healthfulness of their entrées by reducing the use of refined grains, excess sodium, saturated fat, excess sugar, and fried foods, using guideline-driven nutrition recipes and ingredients. Restaurants have the power, if not the responsibility, to promote health and sustainability rather than profits at the high cost of chronic disease and premature mortality.

## Data Availability

Data are available in the Appendix A, without the individual performance of each restaurant.

## Appendix A

Vegan/vegetarian restaurants are indicated in red, and omnivore restaurants with vegan options are in blue.

**State/Country**	**City**	**Restaurant**
Australia	Melbourne	Pho Rolls 62
Australia	Melbourne	Trippy Taco
England	Wimbledon	Cote Brasserie
England	Wimbledon	Parkside Restaurant
England	Wimbledon	Parkside Market
Australia	Melbourne	Moroccan Soup Bar
Australia	East Melbourne	Bedggood & Co
Australia	Melbourne	Vapiano Flinders Lane
France	Paris	Fuga
Australia	Melbourne	Gaylords Indian Restaurant
Australia	Abbottsford	Aviary Melbourne
Australia	East Melbourne	Two Fat Indians (closed)
England	London	Las Iguanas Restaurant
England	London	Hide
England	London	Megan’s
England	London	Cojean
England	London	Thai Girder
Australia	Melbourne	Charcoal Fish
Australia	East Melbourne	Square and Compass
England	London	Gauthier Soho
England	London	Café Laville
England	Wimbledon	Leon’s Naturally Fast Food
England	London	Krua
Germany	Stuttgart	Prince of India
Germany	Mannheim	House of India
France	Paris	Brasserie Lola
France	Paris	Lola Café
France	Paris	Café Victor Hugo
France	Paris	Le Petit Tibet
France	Paris	Café Di Roma
Australia	Melbourne	Terra Rossa Restaurant
Australia	Melbourne	By Korea CBD
Australia	Melbourne	Saigon Pho
Italy	Positano	Palazzo Murat
Australia	Hobart	Burger Got Soul
Australia	Melbourne	Spanish Donut Van
Australia	Melbourne	Hecho in Mexico
Australia	Melbourne	Yeah Boy
Australia	Melbourne	Tadka Hut
Ireland	Killarney	Stonechat
Canada	Montreal	Arepera
Spain	Seville	La Bartola
Spain	Seville	La Gorda de las Setas
Japan	Osaka	55 Plus
Viet Nam	Ho Chi Min City	Anan Saigon
Panama	Panama City	El Jardin Restaurant Cusco
Portugal	Lisbon	#treestory
Czechia	Prague	Amber Kebab Pizza
Netherlands	Amsterdam	Kilamanjaro
Italy	Rome	Rosemary and Timoty Terra e Sapori
Italy	Rome	Caffe Washington
Italy	Rome	The Flann O’Brien
Germany	Berlin	Pho Noodle Bar
Germany	Berlin	Stadtsalat
Slovakia	Bratislava	Brixton House
Slovakia	Bratislava	Urban Bistro
Hungary	Budapest	Szimply
Hungary	Budapest	Akademia Italian
Hungary	Budapest	Franziska Pest
Hungary	Budapest	Japanika
Austria	Salzberg	My Indigo
Germany	Munich	Hofbräuhaus
Germany	Munich	Cotidiano
Switzerland	Stein am Rhein	Pok Thaifood
Germany	Singen	Back Werk
France	Strasbourg	Maison Kammerzell
Ireland	Dublin	Full Moon Thai Restaurant
Scotland	Edinburgh	Makars Mash Bar
Scotland	Fort William	The Crofter
Scotland	Fort William	The Grog and Gruel
Greece	Athens	Ohh Boy
Italy	Florence	Café Dell’Oro
Italy	Rome	Coming Out
Dominican Republic	Punta Cana	Dolce Italia Cap Cana
Mexico	La Paz	Green And Rosse
Spain	Barcelona	Negro Rojo
Spain	Barcelona	Miss Sushi Illa Diagonal
Spain	Barcelona	Buenas migas barcelona
Italy	Sorrento	Bar Monnalisa Ristorante,
Illinois	Niles	HonesT
Oregon	Portland	Bamboo Sushi
Kentucky	Louisville	Shahar Café (old)
Illinois	Bloomingdale	Sarpino’s Pizza
Arizona	Scottsdale	Cooks and Crafts
Illinois	Lombard	Blackberry Market
Arizona	Sahuarita	Pub 1922
Illinois	Des Plaines	American Wild Burger
Kentucky	Louisville	Simply Thai
Kentucky	Louisville	Funmi’s Café
Kentucky	Louisville	Blaze Pizza
California	San Diego	Melting Pot
Illinois	Willowbrook	Mod Pizza
Kentucky	Louisville	Mesh Restaurant
Kentucky	Louisville	Mt. Fuji
Kentucky	Louisville	The Grape Leaf
Indiana	New Albany	Onion Restaurant & Tea House
Virginia	Charlottesville	Brasserie Saison
Hawaii	Honolulu	Tanaka Ramen & Izakaya
Kentucky	Louisville	Panera Bread
Kentucky	Louisville	California Pizza Kitchen
Illinois	Oak Brook	Sweetgreen
Illinois	Oak Brook	The Table at Crate
Ohio	Mason	Chipotle
Kentucky	Louisville	CoreLIfe Eatery
Illinois	Morton Grove	Moretti’s Restaurant
Florida	Miami Beach	Piola Fresh Pizza Restaurant
Illinois	Elmhurst	Kan Ya Ramen and Asian Cuisine
Kentucky	Louisville	J Gumbo’s
Illinois	Hoffman Estates	Taste of Thai
Florida	Kissimmee	Uno’s Pizzeria
Florida	Orlando	Jerusalem
Kentucky	Louisville	Joella’s Hot Chicken
DC	Washington	Sticky Fingers Sweets and Eats
Michigan	Detroit	Lunchtime Global
Illinois	LaGrange	Prasino
Hawaii	Honolulu	Cheesecake Factory
Kentucky	Louisville	Chuy’s
Kentucky	Louisville	McAlister’s Deli
Kentucky	Lexington	Vinagrette Salad Kitchn
Kentucky	Louisville	Molly Malone’s Irish Pub
Kentucky	Louisville	Mayan Café
Kentucky	Louisville	Guacamole Modern Mexican
New York	Flushing Queens	Siam Thai
New York	Flushing Queens	nan-xiang-xiao-long-bao
Florida	Aventura	Pura Vida
Ohio	Warrensville Heights	Café Avalaun
Illinois	Glendale Heights	Buona Beef
Illinois	Chicago	Grand Lux Cafe
Indiana	Indianapolis	Seasons 52
Illinois	South Barrington	Wok n Fire
Ohio	Cleveland	Townhall TH Short North
Ohio	Independence	Melt Bar & Grilled
Kentucky	Louisville	District 6
Florida	Miami Beach	Fresh Garden Bowls
Illinois	Chicago	Blue Sushi Sake Grill
Kentucky	Louisville	Anoosh Bistro
DC	Washington	Open City
Michigan	Kalamazoo	Marushushi
Michigan	Grand Rapids	Rose’s
Michigan	Grand Rapids	Red Bowl
Florida	Lake Nona	Toasted
Illinois	Elmhurst	Currito
Kentucky	Louisville	Lueberry
Kentucky	Louisville	Tandoori Fusion
Kentucky	Louisville	Nam Nam
California	Oceanside	Sabai Sabai Thai
Kentucky	Louisville	Safier Mediterranean Deli
Indiana	Indianapolis	Yard House
Arkansas	Little Rock	The Root Cafe
California	Oceanside	Ocean Thai Cuisine
California	Laguna Beach	Nirvana Grille
California	Laguna Beach	Coyote Grill
California	Laguna Beach	Sapphire
Kentucky	Louisville	Green District
Florida	Winter Park	Mellow Mushroom
Kentucky	Louisville	Noosh Nosh
Illinois	Chicago	NiuB
DC	Washington	Bus Boys and Poets
Kentucky	Louisville	Sala Thai
Illinois	Villa Park	Shahi Nihari & Chopsticks
Kentucky	Louisville	El Mundo Highlands
Kentucky	Louisville	P.F. Chang’s
Kentucky	Louisville	Full Stop Station
Illinois	Chicago	True Food Kitchen
Illinois	Park Ridge	Canton Tea Garden
Kentucky	Louisville	Naive Resturant
Kentucky	Louisville	Redi Zeti Restaurant
Kentucky	Louisville	Eat a Pita
Kentucky	Louisville	Shreeji Indian Restaurant
Hawaii	Honolulu	natuRe Waikiki
Illinois	Glen Ellyn	Blackberry Market
Kentucky	Louisville	Vietnam Kitchen
KentucKy	Louisville	Abyssinia
Illinois	Bensenville	Kitchen Life
Arizona	Scottsdale	Picazzo’s
Illinois	Chicago	Sunda
Arizona	Scottsdale	Protein House
Illinois	Lombard	Bat 57
Kentucky	Louisville	La Que
Indiana	Indianapolis	Axum Ethiopian Resturant
California	Oceanside	Thai Thai
California	Sacramento	Paesanos Pizza
Illinois	Chicago	Fons Empanadas
Illinois	Chicago	Casati’s Modern Italian
Illinois	Chicago	lao sze chuan
Kentucky	Louisville	Funmi’s Café
Kentucky	Louisville	Dragon King’s Daughter
Kentucky	Louisville	Ramsi’s Cafe on the World
Kentucky	Lexington	Carson’s Food and Drink
Arizona	Scottsdale	Pita Jungle
Arizona	Tucson	Selena’s Salvadorian Restaurant
Illinois	Glenview	BonChon
MIchigan	Kalamazoo	600 Kitchen And Bar
MIchigan	Grand Rapids	Gaslight Kitchen
North Carolina	Jacksonville	The Old Siam
Maryland	Chevy Chase	Mei Wah
Maryland	Bethesda	Flower Child
Wisconsin	Eau Claire	Jade Garden
Kansas	Kansas City	Succotash
California	Santa Ana	The Den Café
DC	Washington	Rumi’s Persian Kitchen
DC	Washington	Oyster Oyster
DC	Washington	Beau Thai
DC	Washington	Bibibop
DC	Washington	Boqueria
Oregon	Dundee	Red Hills Provincial
Minnesota	Brainerd	Diamond House
MIchigan	Westland	Anna’s House
Virginia	Alexandria	Virtue Feed and Grain
Kentucky	Louisville	All Thai’d Up
Kentucky	Louisville	Queen of Sheba
Kentucky	Louisville	Abol Ethiopian Coffee
Kentucky	Louisville	Varanese
Kentucky	Louisville	Full Arepa KY
Kentucky	Louisville	Breakfast AF
Kentucky	Louisville	Against the Grain
Arizona	Scottsdale	Caramba Mexican Restaurant
Arizona	Chandler	Dog Haus
Arizona	Chandler	Pokiluv
Arizona	Green Valley	Saigon Flavor
Arizona	Tucson	Lemongrass Eastern Asian
Arizona	Tucson	Hana Tokyo
Arizona	Tucson	Taqueria La Esquina
Arizona	Surprise	Saigon Kitchen
Arizona	Surprise	Tokyo Joe’s
Illinois	Wheaton	Palian Burmese
Illinois	Chicago	Yassa African Restaurant
Illinois	Chicago	The Flying Saucer
Illinois	Chicago	Sweetgreen
Florida	Fort Lauderdale	Fresh First
Florida	Sunny Isles Beach	Treesome Natural Food Café
Georgia	Atlanta	Satto Thai & Sushi
California	Culver City	Phovorite Vietnamese Kitchen
California	Carlsbad	Campfire
Ohio	Montgomery	The Pacific Kitchen
Ohio	Cinncinati	Onolicious Hawaii
Kentucky	Lexington	Pasta Garage
Massachussets	Pittsfield	Dottie’s Coffee Lounge
MIchigan	Grand Rapids	Bankok Taste Cuisine
MIchigan	Grand Rapids	Thai Table
MIchigan	Grand Rapids	Magiamos
MIchigan	Grand Rapids	Wolfgang’s
MIchigan	Royal Oak	Detroit Eatery
MIchigan	Northville	Koji Japanese Restaurant
MIchigan	Northville	Los Tres Amigos
Indiana	New Albany	Kim Bab
Indiana	Jeffersonville	Red Yeti
Michigan	Royal Oak	Ale Mary’s Beer Hall
Virginia	Falls Church/Merrifield	Silver Diner
Indiana	New Albany	Kim Bab
Indiana	Lebanon	Penn Station East Coast Subs
Kentucky	Louisville	Renshoku Ramen
Kentucky	Louisville	Pizza Donisi
Kentucky	Louisville	Chik’n And Mi
Kentucky	Louisville	Chef Shaq Kitchen
Kentucky	Louisville	Smashburger
California	Oceanside	The Lab Collaborative
New York	New York City	Nan Xiang Xiao Long Bao
Georgia	Atlanta	Freshii
Illinois	Chicago	KFC: Beyond Fried Chicken Nuggets.
Illinois	Chicago	Burger King
Illinois	Chicago	Chipotle
Florida	Orlando	Rose and Crown
Florida	Orlando	Selam Ethiopian & Eritrean Cuisine
Wisconsin	Kohler	Imigrant Restaurant
Wisconsin	Kohler	Horse and Plow
Kentucky	Louisville	Jasmine Asian Bistro
Illinois	Hillside	Emillios Tapas Bar
Illinois	Chicago	Wilde Bar And Res
Illinois	Chicago	Gene & Georgetti St
DC	Washington	Tono Sushi
Florida	Orlando	Thai Thani Restauran
Florida	Orlando	Bombay Cafe
California	Palm Springs	Thai Smile Palm Springs
Italy	Sorrento	Bar Monnalisa Ristorante
Ohio	Lexington	Happy House Inc
California	Oceanside	Rim Talay Thai Cuisine
Illinois	Chicago	Nandos South Loop
Illinois	Chicago	Burrito Beach Mexican Grill
California	Woodland Hills	Juicy Ladies
California	Oceanside	Ocean Thai Cuisine
Florida	Lake Buena Vista	Chef Mickey’s
Florida	Kissimmee	So Fresh
Illinois	Chicago	Bad Hunter
France	Paris	Wild & The Moon—Opéra
Australia	Melbourne	Lord of the Fries
Australia	Collingwood	Smith + Daughters
Australia	Melbourne	Union Kiosk
Australia	Kensington	Mesy Burger
Australia	Richmond	Soul Plate Kitchen
France	Paris	Victor and Germain
Australia	Melbourne	Crossways Food for Life
Curacao	Willemstad	De Governeur
France	Antibes	Raw Living
France	Paris	Veg’Anne Restaurant
Australia	Richmond	Fina’s Vegetarian Cafe
France	Paris	Mōpa
England	London	Plants by de
France	Cannes	Fuso
France	Pantain	Le Clairiere
France	Paris	Apeti Segur
Australia	Melbourne	I Luv Vegan
Australia	Melbourne	OM Vegetarian
Australia	Melbourne	Madam K Vegetarialn
Australia	Melbourne	Cookatoo Kitchen
Australia	Melbourne	Gopal’s Pure Vegetarian
Australia	Hobart	Heart Food
England	London	Wulf and Lamb—Marylbone
France	Paris	Copper Branch
France	Paris	Theory Burgers and Bowls
Australia	Melbourne	Red Sparrow Pizza
Australia	Melbourne	Funghi e tartufo
Australia	Anglesea	Diggers Vegie Kitchen
France	Paris	Gourmet Burger—Burger Vegan Arc-en-ciel
France	Paris	Vegan Hero
Germany	Mannheim	Heller’s Vegetarisches
Australia	South Yarra	Jumi’s
Australia	St. Kilda	Sister of Soul
Germany	Munich	Siggis
France	Nice	Panasia
France	Paris	Gentle Gourmet
Australia	Richmond	Tofulicious
France	Nice	Not Dog
Australia	Southbank	Vegie Kitchen
Australia	Melbourne	Vegie Tribe
Germany	Heidelberg	Restaurant ALGE Heidelberg
Monaco	Monte Carlo	Eat Me
Virgin Islands	St. Thomas	Nagee Vegetarian and Ital Catering
Australia	Collingwoord	Las Vegan Café
Australia	Fitzroy	Transformer Fitzroy
Australia	Melbourne	Vegie Bar
Australia	Melbourne	Radhey Kitchen and Chai Bar
Australia	Melbourne	Phuket I’m Vegan
Australia	Melbourne	Vegan Valley
Australia	Melbourne	Gong De Lin
Australia	Fitzroy	Vegie Mum
Australia	Richmond	Easy Vegan
France	Paris	Hank Vegan Burgers
France	Paris	Vegan Folie’s Paris’
France	Paris	Happiz
France	Paris	Brasserie 2eme Art
Spain	Barcelona	Restaurant Vegetalia
Panama	Panama City	Planticeria
Spain	Seville	Balino Yoga Café
Spain	Seville	Ringo Banana Brunch
Spain	Madrid	Mad Madd Vegan
France	Paris	Green Farmers Vegan
England	Manchester	Wawin Vegan Chinese
France	Montrouge	Maison Sūkoon
Saudi Arabia	Jeddah	Vegan Dinosaur
Israel	Tel Aviv	Alegria
Argentina	Rosario	Vrinda
Italy	Amalfi	Natural Experience
Italy	Rome	Il Margutta
Italy	Rome	Ops Cuchina Mediterranea
Italy	Rome	Mater Terrae Restaurant
Italy	Naples	Green Mama Fast Food
Austria	Vienna	Gaia Kitchen
Austria	Vienna	Vegiezz
Indonesia	Jakarta	Vesne Vegetarian Kitchen
Germany	Berlin	Swing Kitchen
Germany	Berlin	Frea Bakery
Chechia	Prague	Linh’s Vegan Corner
Slovakia	Bratislava	Balans Bistro
Hungary	Budapest	Vegan Garden
Austria	Salzberg	The Heart of Joy Café
Austria	Salzberg	The Green Garden
Austria	Salzberg	GustaV
Germany	Munich	Dr Drooly
Germany	Munich	Emmi’s Kitchen (Glockenbach)
Germany	Munich	A Little Lost
Germany	Munich	Prinz Myshkin
Luxembourg	Luxembourg	Nirvana Café
Luxembourg	Luxembourg	Beet Vegan Resturant
Ireland	Dublin	The Saucy Cow
Scotland	Edinburgh	Sora Lella
Scotland	Edinburgh	Black Rabbit
Scotland	Edinburgh	Henderson Restaurant
Scotland	Fort William	The Wildcat Café
Italy	Rome	Universo Vegan
Chechia	Prague	Palo Verde
India	Mumbai	Aharveda Vegan and Beyond
Kuwait	Kuwait City	The Hungry Vegan
Egypt	Alexandria	Koshary Ala Sokhon
Brazil	Sao Paulo	SuBte Vegan
Cuba	Havana	Shamuskia
France	Montrouge	55 Plus
Mexico	Cancun	Herbivoro
England	London	222 Vegan Bowl Food
Mexico	La Paz	Capuchino
Spain	Madrid	Rayen Vegano
Maylasia	Kuala Lumpur	Just Life Vegan Café
California	Irvine	Wheel of Life Vegan Restaurant
California	San Diego	Peace Pies
Illinois	Chicago	Top Notch Vegan Burgers
Kentucky	Louisville	VeganVille
Pennsylvania	Philadelphia	Blackbird Restaurant
California	San Diego	Loving Hut
Kentucky	Louisville	V-Grits
Canada	Brampton	Odd Burger
Canada	Toronto	Kupfert and Kim
Canada	Toronto	Sorry I’ve Got Plants
Canada	Toronto	Fresh Kitchen and Juice Bar
Louisiana	New Orleans	Seed Vegetarian
Kentucky	Louisville	Manna Kitchen
Illinois	Chicago	Tawa Pulav
DC	Washington	Zoe’s Vegan Delight
DC	Washington	Ahra Cafe
Kentucky	Louisville	Against the Grain
Illinois	Bloomingdale	Pho-licious
Indiana	Indianapolis	Three Carrots
Oregon	Portland	Fortune Plant Based Papi
Kentucky	Louisville	Heart and Soy
Massachussetts	Cambridge	Plant Pub
Louisiana	New Orleans	Kindred
Kentucky	Louisville	Soul Hi
Arizona	Scottsdale	UNIQ Burger
Pennsylvania	Pittsburgh	Allegro Bakery
Pennsylvania	Pittsburgh	Shadobeni Trinidad
Arizona	Scottsdale	Early Bird Vegan
California	San Diego	Cafe Gratitude
California	Dana Point	Eden Café
Ohio	Cleveland	Cloak and Dagger
Ohio	Cleveland	Cleveland Vegan Café
England	Chelsea	Wulf and Lamb Chelsea
Indiana	Indianapolis	Plantastic Indy
Oregon	Portland	Blossoming Lotus
Oregon	Portland	Next Level Burger
California	Indian Wells	Veggie Grill
Illinois	Hoffman Estates	Annapurna Simply Vegetarian
New York	New York City	Blossom
New York	New York City	Bodhi Village Kosher Vegetarian
Illinois	Orland Park	Can’t Believe It’s Not Meat
Michigan	Grand Rapids	Forty Acres
Hawaii	Honolulu	Aloha Bowls
California	Pasadena	My Vegan Restaurant
DC	Washington	DC Vegan
Illinois	Chicago	Blind Faith Cafe
DC	Washington	Shouk
DC	Washington	Equinox
DC	Washington	Equinox on 19th
Hawaii	Honolulu	Yoga Under the Palms Hawai‘i
DC	Washington	Hip City Vegan
Arizona	Scottsdale	Green New American Vegetarian
Kentucky	Louisville	Half Peach Bakery
Arizona	Phoenix	Vegan House
Arizona	Phoenix	The Coronado Phoenix
Arizona	Phoenix	Lowdown Love Vegan
Arizona	Phoenix	Earth Plant Based Cuisine
Florida	Miami Beach	Plnthouse
Illinois	Chicago	Alice and Friends Vegan Kitchen
Illinois	Glenview	Alice and Friends at the Glen
California	Anaheim	Thuyền Viên Vegetarian Restaurant
Illinois	Chicago	Amitabul
Illinois	Chicago	Healthy Substance
New York	New York City	Dirt Candy
New York	New York City	Plant Junkie
New York	New York City	Candle 79
California	Oceanside	The Plot
Illinois	Chicago	Althea
Arizona	Scottsdale	Fresh Mint
California	Los Angeles	Beyond Vegan Eats
California	Los Angeles	Sun Cafe
Illinois	Oak Park	Munch
Illinois	Winnetka	Spirit Elephant
Iowa	Iowa City	Trumpet Blossom
Illinois	Chicago	Bloom
Illinois	Chicago	Planta Queen Chicago
Arizona	Phoenix	Vegan and Vines
Arizona	Phoenix	Infruition
Arizona	Phoenix	Goji Berry
Illinois	Chicago	Native Foods
Hawaii	Honolulu	Tane Vegan Izakaya
Arizona	Tucson	Beaut Burger
Illinois	Chicago	Loving Heart
Illinois	Chicago	Chicago Diner
Texas	Fort Worth	Belenty’s Love
Kentucky	Louisville	Roots
Kentucky	Louisville	Cosmic Bird
Indiana	Indianapolis	Black Leaf Vegan
DC	Washington	Fruitive
DC	Washington	Bubbie’s Plant Burgers
Florida	Miami Beach	Plant Theory
Florida	Orlando	Earthy Picks
Florida	Lake Nona	Veg’n Out
Florida	Orlando	Leguminati
Florida	Orlando	The Madras Café
Florida	Orlando	Khasiyat
Illinois	Niles	HonesT
Illinois	Chicago	Upton’s LIberation Cafe
Illinois	Chicago	Arya Bhavan
Pennsylvania	Philadelphia	Vedge
Illinois	Chicago	Vegan Plate
Hawaii	Honolulu	Peace Cafe
New York	Flushing	Green Zephony Vegan Restaurant
Illinois	Chicago	Chicago Raw Food
Illinois	Chicago	Vegan World Café Catering
Illinois	Chicago	Karyn’s Fresh Corner
Illinois	Chicago	Karyn’s Cooked
Illinois	Chicago	Karyn’s on Green
Illinois	Flossmoor	Karyn’s Kitchen
Illinois	Lockport	The Vegan Café
Pennsylvania	Westport	Goodbeet
Illinois	Palos Heights	Meeks Vegan Kitchen
California	Laguna Beach	Zinc Café
California	Huntington Beach	Mitasie
California	Lake Forest	Mitasie Café
California	Los Angeles	Real Food Daily Café
California	Los Angeles	The Wheel of Life Vegetarian Thai Cuisine
California	Agoura Hills	Ma Kim Vegan
California	Santa Ana	La Vegana Mexicana
Illinois	Buffalo Grove	Melted
North Carolina	Raleigh Durham	Elements Gastropub
Virginia	Alexandria	Plnt Burger
Michigan	East Grand Rapids	Little Africa Ethiopian Cuisine
Illinois	Chicago	Soul Veg City
New Jersey	Westmont	Heart Beet Kitchen
Georgia	Atlanta	Soul Vegetarian
Georgia	Atlanta	Slutty Vegan
Georgia	Atlanta	Cafe Kulture
Georgia	Atlanta	Herban Fix
Georgia	Atlanta	Hippie Habachi
Georgia	Atlanta	La Semilla Reynoldstown
Georgia	Atlanta	Planta Atlanta
Georgia	Atlanta	Full Taste Buckhead
Illinois	Chicago	Kal’ish (closed)
Illinois	Chicago	Majani Vegan Soulful Cuisine
Illinois	Chicago	Handlebar
Illinois	Chicago	Veggitopia
Illinois	Chicago	Kale My Name
Illinois	Chicago	Kitchen 17
Illinois	Chicago	Elephant + Vine Vegan
Illinois	Chicago	Art of Dosa
Indiana	Indianapolis	Fever’s Plant Based Kitchen
DC	Washington	Fare Well
DC	Washington	Pow Pow
DC	Washington	Elizabeth’s Gone Raw
Virginia	Herndon	GreenFare Organic
Michigan	Detroit	Nosh Pit Detroit
Illinois	South Holland	Daisy’s Vegan Catering
Alabama	Madison	The Veggie
Tennesee	Nashville	Nashville Sunflower Cafe Berry Hill
Arizona	Chandler	Nana’s Kitchen Soul
Arizona	Tucson	Lovin’ Spoonfuls
Arizona	Tucson	Govinda’s Natural Food Buffet
Arizona	Phoenix	Veggie Village
MIchigan	Royal Oak	Cacao Tree Café
MIchigan	Royal Oak	Modern Vegan
MIchigan	Ann Arbor	Detroit Street Filling Station
MIchigan	Farmington	Treehouse for Earth’s Children
MIchigan	Novi	Tumerican Vegetarian Cuisine
Texas	Austin	Nori Austin
MIchigan	Farmington	Chutneys Indian Vegetarian Cuisine
Michigan	Ann Arbor	Seva Detroit
Pennsylvania	New Hope	V-Spot Food
Wisconsin	Stevens Point	Wicked Willow Cafe
Wisconsin	Milwaukee	Strange Town
South Carolina	Charleston	Neon Tiger
Kentucky	Louisville	Shahar Vegan Cafe
New York	New York City	Eleven Madison Park
New York	New York City	Beyond Sushi
New York	New York City	Fermento
